# Electron Beam Irradiation Induced Multiwalled Carbon Nanotubes Fusion inside SEM

**DOI:** 10.1155/2017/8563931

**Published:** 2017-10-23

**Authors:** Daming Shen, Donglei Chen, Zhan Yang, Huicong Liu, Tao Chen, Lining Sun, Toshio Fukuda

**Affiliations:** ^1^Robotics and Microsystem Center, Soochow University, Suzhou 215006, China; ^2^School of Mechatronics Engineering, Harbin University of Science and Technology, Harbin 150080, China; ^3^Department of Micro-Nano Systems Engineering, Nagoya University, Nagoya 464-0814, Japan

## Abstract

This paper reported a method of multiwalled carbon nanotubes (MWCNTs) fusion inside a scanning electron microscope (SEM). A CNT was picked up by nanorobotics manipulator system which was constructed in SEM with 21 DOFs and 1 nm resolution. The CNT was picked up and placed on two manipulators. The tensile force was 140 nN when the CNT was pulled into two parts. Then, two parts of the CNT were connected to each other by two manipulators. The adhered force between two parts was measured to be about 20 nN. When the two parts of CNT were connected again, the contact area was fused by focused electron beam irradiation for 3 minutes. The tensile force of the junction was measured to be about 100 nN. However, after fusion, the tensile force was five times larger than the tensile force connected only by van der Waals force. This force was 70 percent of the tensile force before pulling out of CNTs. The results revealed that the electron beam irradiation was a promising method for CNT fusion. We hope this technology will be applied to nanoelectronics in the near future.

## 1. Introduction

The gate scale of the transistor of integrated circuit (IC) chip is down to 10 nm. With the introduction of sub-10 nm transistor, the scaling trend of transistor with silicon eventually reaches its physical limitation [1]. The quantum chipping effects become more prominent as the length of channel between source and drain was reduced to several nanometers. With photolithography and advanced ultraviolet etching processing technology, central processing unit (CPU) and graphic processing unit (GPU) were integrated within hundreds of billions of transistors. They reduced the processor's thermal power consumption and enhanced the processor frequency significantly [2]. In recent years, carbon nanotube, which was discovered by Raghavan in 1991 [3], attracted great interest of not only researcher but also manufacturing engineer on a conductive channel for the transistor less than 10 nm. The multiwalled carbon nanotube consisted of rotation of individual graphene sheets with respect to the needle axes [4]. Carbon nanotubes were noticed greatly because of their exceptional electrical, mechanical properties and unique electronic transport characteristics [5, 6]. It is expected that these properties will be used by employing carbon nanotubes as structural or electrical components. Some major companies in the world such as IBM Corporation [7] and Intel Corporation [8] proposed a new process of carbon nanotube. CNT had a similar molecular structure to the graphene, which consisted of a hexagonal lattice of carbon atoms [9]. The carriers in the CNT can move freely in each graphene sheet because of ballistic transport [10]. It is on the order of 10^9^ A/cm^2^, which is three orders of magnitude larger than Cu [11]. The current density of CNT field-effect transistor (FET) was four times more than that of the best silicon devices. In addition, it was performed at a low operating voltage. With the scale of transistors becoming smaller, the switching speed will be improved significantly [12]. Copper as the traditional conducting material was more vulnerable to electromigration damage [13]. Copper resistivity increases due to electron scattering at the surface. By this transport form of electrons, the current density of carbon nanotube was two or three orders of magnitude higher than that of Cu [14], making CNT an ideal material for nanodevice and electronic circuits.

The carbon nanotube interconnection technology is a crucial part for structure manufacture and functional device preparation and assembly. The quality of the connection directly determines the reliability of the functional device. The existing interconnection methods include chemical vapor deposition (CVD), high energy beam irradiation technology, arc discharge, and ultrasonic vibration interconnecting technology [15]. During the conventional processing, it was not possible to realize valid pick-up and alignment of carbon nanotube in three-dimensional space. Besides, carbon nanotubes were not interconnected by appropriate methods. To solve these problems, some scholars have designed and developed the nano operating system. Fukuda et al. constructed a nanorobotic manipulation system consisting of 4 operation units with 16 DOFs [16]. This system can be used for nanomanipulation and nanoassembly. Ru et al. demonstrated a 4-probe automated nanomanipulation system inside an SEM for a nanomanipulation task [17]. In IC manufacturing, CNT can be placed through boom-up technology [18]. It can effectively solve the problem which the top-down fabrication processing is faced with.

Over these years, CNT was proven to have potential application in large-scale integration interconnection. Nanometer-scale electronic device has been realized and widely applied in computer chips, tiny wires, and so on by many interconnection methods of carbon nanotubes [19]. Over the decades, the nanodevices [20–22] have made great breakthrough due to interconnection technology. The interconnecting technology has become the key component in nanodevices manufacture. Wu's Group successfully welded double-walled CNTs inside the vacuum tube by vacuum brazing of CNTs with a eutectic alloy (AgxCuy) doped with Ti. The interconnection process needed low cost and the contact resistance was low [23]. Krasheninnikov et al. performed MD simulations of ion irradiation induced CNT welding [24] and showed how this approach could be used to solder CNTs. However, the energy was consumed with time going on. Chen and Zhang showed that the focused electron beam in a scanning electron microscope (SEM) can be used to deposit a small amount of hydrocarbon contamination so as to attach the tubes on an AFM tip [25]. The adhesion was large enough that the CNT was attached on the AFM tip firmly. However, the experimental devices were contaminated during the interconnection process. Peng et al. synthesized branching structures of H-junctions and multiple Y-junctions CNT using a thermal chemical vapor deposition method [26]. The spatial resolution, flexibility, and controllability of welds between individual nanowires and nanoobjects were improved radically by this method.

Fedorov et al. applied focused-electron-beam-induced capabilities to fuse CNT with electrode [27]. It would have a direct positive impact on enhancing functionality, improving quality, and reducing fabrication costs for electronic devices. These methods destructed the properties of CNTs to some degree. It is introduced that the nanotubes are connected by the fusion C-C bonds and the interconnection was stronger than the previous CNTs connected without fusion. What is more, this method has some advantages over any other methods [28]. The carbon nanotube interconnecting technology has no contamination. Nanodevices can be in mass production with interconnecting carbon nanotubes [29]. There is no need of other materials during the process of fusing the C-C bond with electron beam [30]. Study has shown that the carbon nanotubes can be connected at any angle and integrated into complex constructions by nanomanipulation [31]. This carbon nanotube interconnection technology can advance the development of small-scale device. However, the carbon nanotubes were not interconnected precisely. These methods changed the surface resistance of interconnected carbon nanotubes. These involved issues were not solved effectively by the above-mentioned interconnection methods.

**Figure 1:**
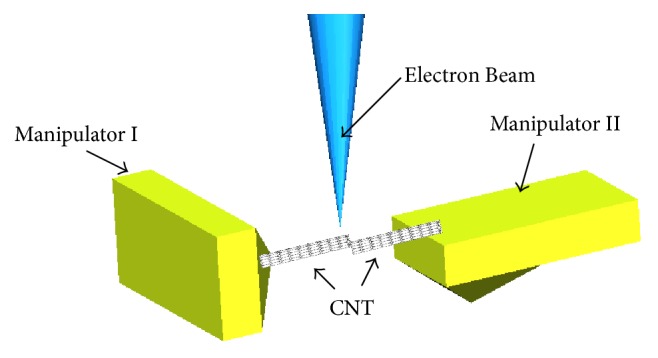
The schematic diagram of fusion method by electron beam irradiation.

This paper presented a method to interconnect carbon nanotube with electron beam to fuse the C-C bond. The carbon nanotubes were picked up and aligned effectively by nanomanipulators. With the development of semiconductor technology, the size of nanodevices is getting smaller and smaller. Effective interconnection of semiconductors has become a major challenge. In future industrial application, large quantities of carbon nanotubes can be fused by electron beam irradiation. The mass production will be achieved by this method. The whole operations were finished in the scanning electron microscope (SEM). We designed three experimental categories to compare fusion effect. In order to study the influences of the irradiation time and the magnification of observation on fusion, the deflection of the AFM should be observed and recorded seriously during the experiment. The effects of these factors were figured out, respectively, through the curves obtained in the experiment.  Figure 1 shows a schematic diagram of fusion method by electron beam irradiation of the MWCNTs.

## 2. Experimental Set-Up

**Figure 2:**
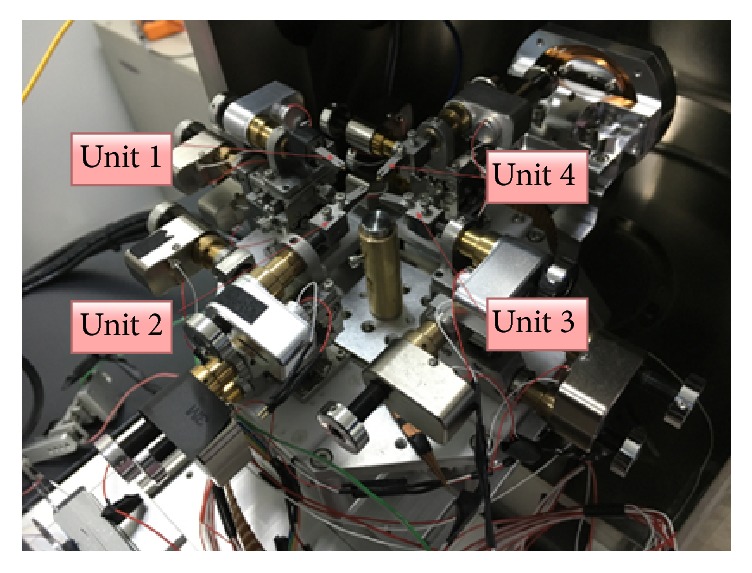
An image of the nanorobotics manipulation system.

The experimental system was designed to study these issues in the SEM.  Figure 2 shows the configuration of the system set-up for nanorobotics manipulation with 21 DOFs. The SEM (Zeiss, MERLIN Compact, resolution: 1.5 nm) was introduced to observe the whole nanomanipulation process that was conducted in the vacuum chamber of the SEM (Table 1). In this system, Unit 1, Unit 2, and Unit 3 were used to operate the nanomanipulation. Unit 1 and Unit 2 (SmarAct, SLC-1720-s) with a resolution of 1 nm shown in Figure 2 were four-axe micromanipulators. Unit 3 consisted of the Picomotor (New Focus, 8301-UHV) and a three-dimensional micromotion stage (Sigma, TSDS-255C) with a resolution of 30 nm to move the CNT bulk. The grippers were designed to fix the AFM cantilever (Olympus, OMCL-TR400PB-1). The AFM was applied to pick up carbon nanotubes from CNT bulk. The carbon nanotubes that were fixed on the AFM cantilever tip were driven by a manipulator. And the moving step of the AFM was set at 5 nm step.

The procedure of the experiment was shown as follows.

**Figure 3:**
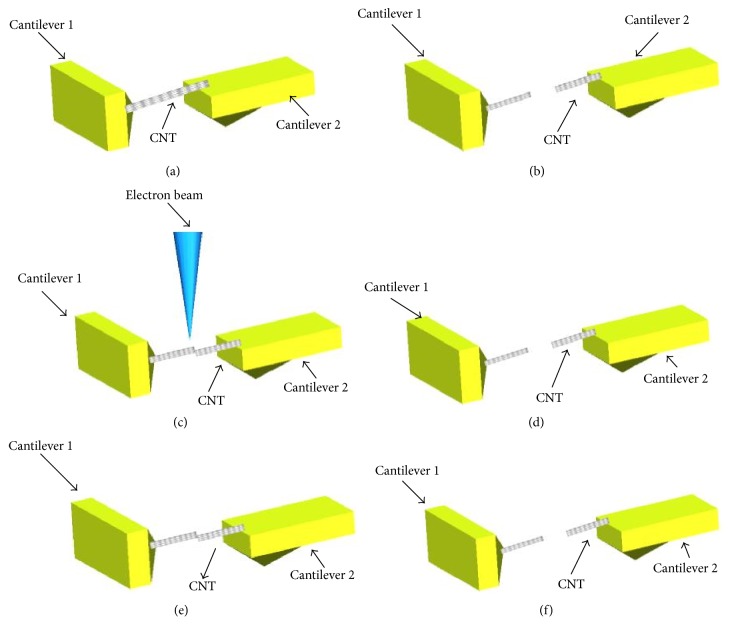
(a) The original position of the CNT. (b) Pulling the CNT until the nanotube breaks. (c) Fusing the CNT. (d) Pulling the CNT into two parts. (e) Connecting CNTs again without fusion. (f) Pulling the CNTs separated apart.

The first step was to pick up a MWCNT. The picked up carbon nanotube was fixed on cantilever 2 as shown in Figure 3(a) and the distance between the two ends of the carbon nanotube was measured.

The second step was to connect the MWCNTs. Cantilever 2 was driven rightwards. The MWCNT was pulled into two parts. The two ends of the distance were recorded when the MWCNT was broken into two parts as shown in Figure 3(b). During the pulling process, the MWCNT was separated into two parts. After the pulling, the total length of the two parts was beyond the previous MWCNT.

The third step was to fuse the MWCNTs. Cantilever 2 was moved with 10 nm step to connect two parts of the CNT. The manipulators stopped moving when the two breaking points of the CNT touched each other. The joint was fused by electron beam irradiation as shown in Figure 3(c). The accelerating voltage was applied to 5 kV and the beam current was 30 pA. The C-C bond of the two MWCNTs where they were in contact together was fused by the electron beam. Two MWCNTs were irradiated for 3 minutes and the magnification was kept at 5000. Before breaking, we recorded the distance of the two joints on the AFMs.

The fourth step was to pull the MWCNTs again. The deflection of cantilever 1 was recorded when the fused MWCNTs broke up as shown in Figure 3(d).

The fifth step was to connect again. The connection force was van der Waals force as shown in Figure 3(e). As soon as they connected, the extra high tension (EHT) was set off for 3 minutes for comparison with the CNT which was fused by electron beam irradiation.

In the following procedure, the connected MWCNTs were pulled again and the deflection of cantilever 1 was recorded when the MWCNTs separated apart.

## 3. Experimental Result

**Figure 4:**
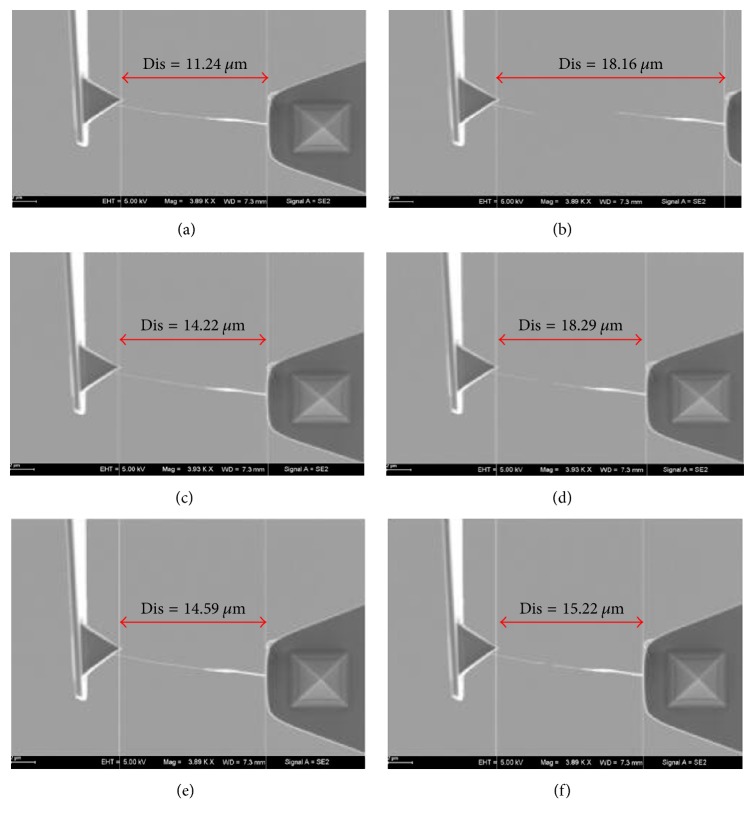
(a) The original distance of two ends of the CNT. (b) The distance when the CNT was dragged. (c) The distance of two ends of the fused CNT. (d) The distance when the fused CNT was dragged. (e) The distance of interconnecting the dragged CNT. (f) The distance of dragging the interconnected CNT.

As shown in Figure 4(a), the MWCNT picked up from the bulk was set on the two manipulators (cantilever 1 and cantilever 2). In the SEM, the length of the MWCNT in the beginning of the experiment was measured at 11.81 *μ*m and the diameter was 39.46 nm. The original distance of the two joints was 11.24 *μ*m. In Figure 4(b), the MWCNT was dragged into two parts and the lengths of the parts were 3 *μ*m and 13.7 *μ*m, respectively. The distance between the two joints was 18.16 *μ*m. The tensile force was calculated to be 140 nN. As Figures 4(c) and 4(d) show, the MWCNT was fused by electron beam irradiation. After that, the fused MWCNT was pulled off again and van der Waals force was calculated to be 12.6 nN. In Figures 4(e) and 4(f), the dragged MWCNT was interconnected and dragged again. When the CNT was interconnected and dragged, the distances of the two ends were 14.22 *μ*m and 18.29 *μ*m, respectively. It was found that the tensile force after fusion was 81.4 nN, which was clearly larger than van der Waals force.

## 4. Discussion

The electron beam was emitted by the electron gun in the SEM irradiated at the joint of MWCNTs. This synthesis was controlled by the systems operating with atomic-scale precision which enabled positional selection at the desired place precisely. The dragged C-C bonds were at the end of the MWCNTs. Because of the existence of the interatomic repulsive force, the fractured C-C bonds could not recover, when the dragged MWCNTs were interconnected. However, the electronic beam transferred to the orbital electrons of the carbon atoms. When the emitted electrons strike the joint, quantum photons generated from the electrons transferred energy to the low-energy orbit electrons. The low-energy orbit electrons would transit to the high-energy orbit; then their vibration frequency and range of the motion were increased. Under this circumstance, the possibility of C-C bonds formation was increasing which promoted lattice reconstruction of carbon atoms, so the properties would be the same as initial CNTs. Before this experiment, van der Waals force was calculated theoretically according to the following formula [28]:

(1) $$\begin{array}{c} W=\frac{A_{s}\pi C\rho_{1}\rho_{2}}{12D^{2}}, \end{array}$$

where *C* is the coefficient in the atom-atom pair potential; *ρ*_1_ and *ρ*_2_ are the numbers of atoms per unit volume in the interaction material. *D* is 0.34 nm, which is the vertical distance of the attractive van der Waals force. *A* is the Hamaker constant between nanotubes [28]:

(2) $$\begin{array}{c} A=\pi^{2}Cp^{2}=2.842\times 10^{-20}\;J. \end{array}$$

According to the tensile force formula, the force was calculated by Hooke's law [32]:

(3) $$\begin{array}{c} F=kd. \end{array}$$

By comparing tensile force and van der Waals force, the tensile force was evidently larger. This result showed clearly that the fusion of carbon nanotubes could increase the tensile force significantly.

**Figure 5:**
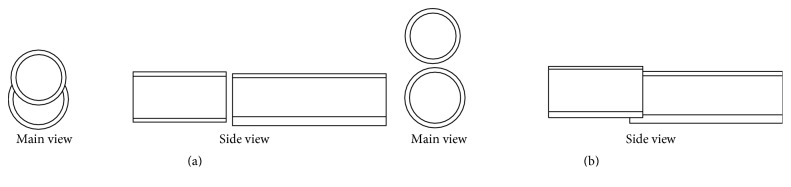
(a) Cross-sectional view and overhead view of head to head configuration of two carbon nanotubes. (b) Cross-sectional view and overhead view of side to side configuration of two carbon nanotubes.

Assuming that the carbon nanotube was a multilayered cylinder, there were two interconnection ways. One was head to head configuration and the other was side to side configuration, as shown in Figures 5(a) and 5(b).

Figure 5(a) shows the head to head configuration. In this case, the centres of the two carbon nanotubes were connected and van der Waals force was the largest. The layers of the carbon nanotubes were 58. The cross-sectional area can be calculated by the following relation equations [33]:

(4) AS=∑n=1n=58 =π0.34+0.035+0.34n2−π0.34−0.035+0.34n2=138 nm2.

According to the calculation, the area is 138 nm^2^. The value of the force was calculated to be 5.3 nN.

The other way of carbon nanotubes interconnection was side to side configuration. The strongest connection force was contained by the following equation [34]. The distance between two outer walls was 0.34 nm, *s* was the interfacial shear stress of nanotubes, which was 2 MPa, and *w* was the contact width of the MWCNTs, which was 100 nm.

(5) $$\begin{array}{c} F_{v}=swe_{x}=6.8\;nN. \end{array}$$

**Figure 6:**
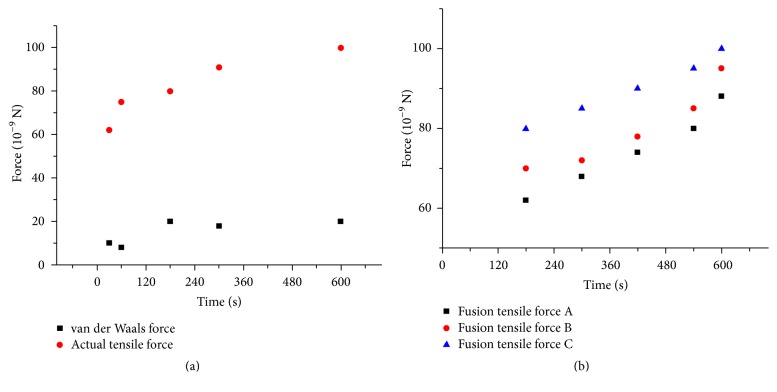
(a) *F*-*T* scatter diagram of actual tensile force and van der Waals force. (b) *F*-*T* curve of actual tensile force of different diameters of CNTs.

First, the two ends of MWCNTs were irradiated for some time. The deflection of the cantilever was tested after dragging the connected nanotube until the new joint broke. After connection, the extra high tension was shut down. When dragging the connected MWCNT, the extra high tension was turned on. The relationship between the forces and time was presented by the *F*-*T* scatter diagram in Figure 6(a). As irradiating time passed by, van der Waals force changed a little, demonstrating that the actual van der Waals force had nothing to do with the irradiating time.

After fusion, the actual tensile force was recorded. It obviously reflected the fusion effect by the *F*-*T* scatter diagram that was shown in Figure 6(a). The tensile force was significantly larger than van der Waals force. What is more, the longer the irradiation time was, the larger the actual tensile force was.

In order to rule out the fortuity, the experiment was divided into several groups by manipulating different diameters of CNTs to repeat the fusion and measure the fusion effect by deflection of AFM. The *F*-*T* scatter diagram of fusion tensile force was shown in Figure 6(b). The fusion effects were almost similar in three different MWCNTs. The electronic beam irradiation methods possessed universality in fusing graphene structure.

**Figure 7:**
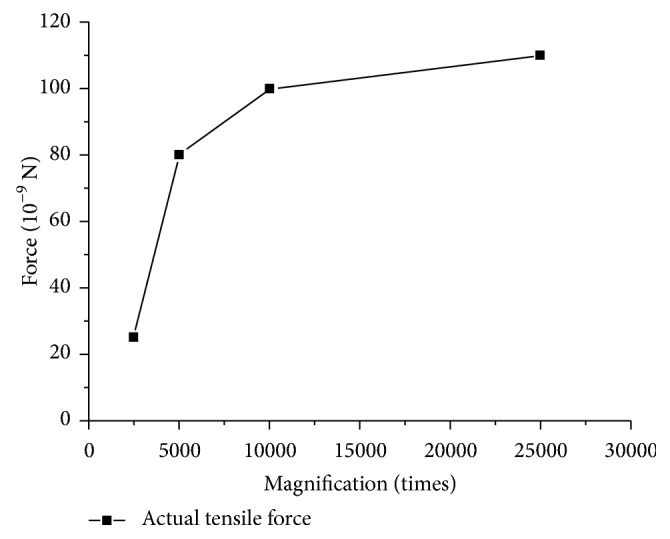
Force and magnification relationship curve of actual tensile force at different magnification times.

Another experiment was designed to verify the effects of different magnification on fusion. The irradiation time was set to 2 minutes under different magnification. The *F*- *M* curve was obtained by experiment. From Figure 7, the force became larger with the increase of magnification. When the magnification increased, the region of observation became small with more electrons gathering. In this condition, more electrons emitted by electronic gun hit extranuclear electrons of carbon atom. The experiment demonstrated that a larger magnification had a better effect on fusion of carbon nanotubes.

**Figure 8:**
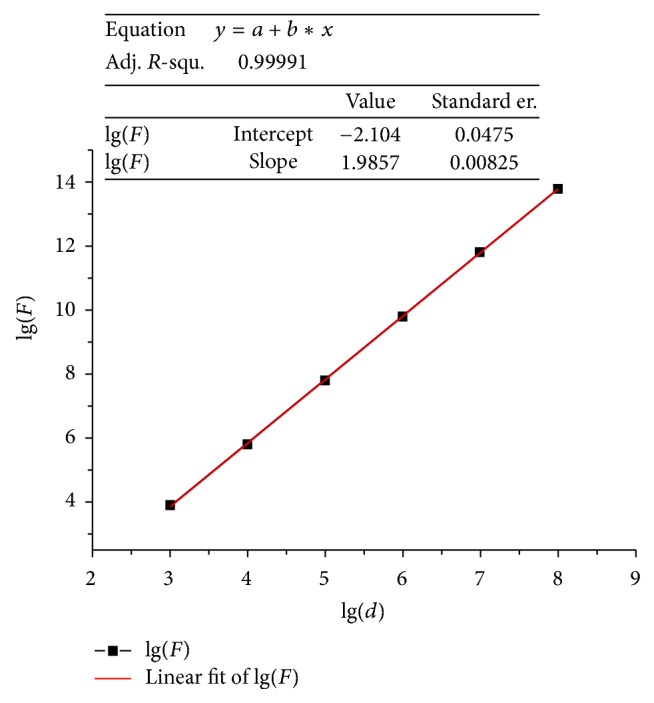
lg(*F*) and lg(*d*) relationship curve of electric field imaging force of CNTs with different diameters.

It was observed that the force becomes larger with the magnification increasing. This phenomenon may be caused by the electric field imaging force. Thus, we calculated the force according to the formula and made the following chart [35]:

(6) $$\begin{array}{c} lg\left(\;F_{ei}\;\right)=lg\left(\;\frac{\pi }{4\varepsilon_{0}}\frac{\varepsilon -\varepsilon_{0}}{\varepsilon +\varepsilon_{0}}d^{2}\sigma^{2}\;\right), \end{array}$$

where *d* is the diameter of the sphere, *ε*_0_ is dielectric coefficient of circumstance, *F*_ei_ is the electrostatic force by electric imaging, and *ε* is the dielectric coefficient of the nanotube. *ε*_0_ = 8.85 × 10^−12^ [F/m]; *ε* = 5*ε*_0_, *σ* = 26.5 [*μ*C/m^2^].  Figure 8 indicates the relationship between the electrostatic force and the diameter of carbon nanotube. The logarithm was adopted here so that linear calculations of the force were convenient. The electrostatic force of carbon nanotube with dozens of nanometers could be calculated by this graph of a function “lg(*F*) = 1.98lg(*d*) − 2.10.” After calculation, we found that the diameter of the CNT was dozens of nanometers and the force was about 11 nN, while the fusion force was about 100 nN. The scale of the force was not an order of magnitude with the fusion force. Therefore, the force could be ignored. When the magnification time increased, the observation region became small with more electrons gathering. In summary, the force was the fusion force.

The fusion method of electronic beam irradiation was reliable. The fusion effect achieved 70 percent which was stronger than other methods and did not damage the original structure. The electron beam will indeed induce hydrocarbon. However, our experimental vacuum order was 10^−4^ Pa. At that point, the free path of hydrocarbon molecules was very large. The distance was calculated at several hundred meters theoretically. But the diameter of carbon nanotube was tens of nanometers. The amount of hydrocarbon accumulation absorbed on the surface of carbon nanotube was very small [36]. Therefore, we think that the hydrocarbon absorbed on the surface of carbon nanotubes can be neglected. The fusion point and location would be controlled.

## 5. Conclusion

This paper proposed a new method of the interconnection of carbon nanotubes. The tensile force of the fused carbon nanotubes was larger than van der Waals force. A C-C bond was newly generated because of the fusion by electron beam irradiation. This interconnection method can connect different-scale carbon nanotubes. Some small-scale electronic devices can be built by nanomanipulation with the development of technology. This method used to interconnect MWCNTs is sturdy because the actual tensile force increased only from 20 nN to 100 nN. In the future work, more efforts will be devoted to interconnect the MWCNTs by arbitrary angles to fabricate nanotransistor.

## Figures and Tables

**Table 1:** Parameters of each nanorobotics manipulator.

Parameters	Unit 1	Unit 2	Unit 3	Unit 4
Model	SLC-1720-s/8301-UHV	SLC-1720-s/8301-UHV	TSDS-255C/8301-UHV	TSDS-255C/8301-UHV
Dimensions (mm)	33*∗*33*∗*30.5/63.5*∗*32.2*∗*56.5	33*∗*33*∗*30.5/63.5*∗*32.2*∗*56.5	66*∗*66*∗*45/63.5*∗*32.2*∗*56.5	66*∗*66*∗*45/63.5*∗*32.2*∗*56.5
Travel (mm)	*X* ± 6, *Y* ± 6, *Z* ± 6	*X* ± 6, *Y* ± 6, *Z* ± 6	*XY* ± 3, *Z* ± 3	*XY* ± 3, *Z* ± 3
Rotate	−360°~+360°	−360°~+360°	−360°~+360°	−360°~+360°
Linear resolution	1 nm	1 nm	30 nm	30 nm
Rotate resolution	<1 microrad	<1 microrad	<1 microrad	<1 microrad
